# Unilateral acute posterior multifocal placoid pigment epitheliopathy (APMPPE) with delayed contralateral eye involvement

**DOI:** 10.1186/s12886-023-03221-8

**Published:** 2024-01-09

**Authors:** Emanuel Mordechaev, Gabriel Shakarov, Deep Parikh

**Affiliations:** New York Eye and Ear Infirmary of Mount Sinai, 310 E 14th St, New York, NY 10003 USA

**Keywords:** White dot syndrome, APMPPE, Uveitis, Ocular inflammation, Placoid

## Abstract

**Background:**

Acute posterior multifocal placoid pigment epitheliopathy (APMPPE) is a rare presumed inflammatory chorioretinopathy characterized by creamy, yellow-white placoid lesions at the level of the retinal pigment epithelium (RPE). Unilateral cases often have fellow eye involvement within days to a few weeks. This report details a rare case of delayed contralateral APMPPE, in which unilateral lesion resolution was followed by contralateral eye involvement 31 months later.

**Case presentation:**

A 38-year-old woman presented with three days of blurry vision and photopsias in the right eye (OD). She endorsed a viral GI illness one month prior. Visual acuity was 20/25 -2 OD and 20/20 -1 in the left eye (OS). Examination revealed creamy, yellow-white placoid lesions in the posterior pole. Fluorescein angiography (FA) was notable for early hypofluorescence and late hyperfluorescence of the lesions, consistent with APMPPE. MRI and MRA brain were negative for cerebral vasculitis. She was treated with oral prednisone with complete resolution of her symptoms, vision, and lesion regression. She then presented 31 months later, with blurry vision OS and similar new creamy, yellow-white placoid lesions in the posterior pole OS. She endorsed receiving an influenza vaccine one month prior. FA again was notable for early hypofluorescence. She was diagnosed with APMPPE, this time involving the left eye, and was once again started on oral steroids with complete resolution. She denied any neurologic symptoms.

**Conclusions:**

APMPPE is an inflammatory vasculitis of the choroid, leading to hypoperfusion and ischemic injury of the RPE with subsequent lesion formation. APMPPE may be preceded by a viral prodrome or vaccination, both of which were seen in this case. Choroidal inflammation seen in APMPPE is therefore thought to stem from immune-mediated processes. Unilateral cases often have fellow eye involvement within days to a few weeks. Single eye involvement with delayed contralateral presentation, as seen in our patient, is rare. This case demonstrates that lesion resolution in one eye can be followed by contralateral eye involvement up to 31 months later, highlighting the importance of routine ophthalmic monitoring for patients with unilateral APMPPE.

## Background

Acute posterior multifocal placoid pigment epitheliopathy (APMPPE) is a rare presumed inflammatory chorioretinopathy described first by Gass in 1968 [1], with an estimated incidence of 0.15 cases out of 100,000. It is characterized by creamy, yellow-white placoid lesions at the level of the retinal pigment epithelium (RPE) [2, 3]. Placoid lesions detected on examination are more precisely delineated with fluorescein angiography (FA) and indocyanine green angiography (ICGA). APMPPE typically presents bilaterally, affects men and women equally, and usually occurs during the second through fourth decades of life. Visual prognosis for APMPPE is generally favorable and it is thought to be a self-limiting condition, but foveal involvement, neurological symptoms, and atypical features such as onset > 60 years old and unilaterality confer worse prognosis [4, 5]. Cases that present unilaterally often tend to involve the contralateral eye within days to a couple of weeks [6].

We report a rare case in which unilateral APMPPE treated with systemic steroids was followed by fellow eye involvement 31 months later. This is the longest documented interval between unilateral presentation of APMPPE and the development of contralateral eye involvement.

## Case presentation

A 38-year-old woman presented with three days of blurry vision and photopsias in the right eye (OD). The patient denied any other ocular or neurologic symptoms. She endorsed a viral GI illness one month prior. Her nausea, vomiting, myalgias, and high fevers had since resolved.

**Fig. 1:**
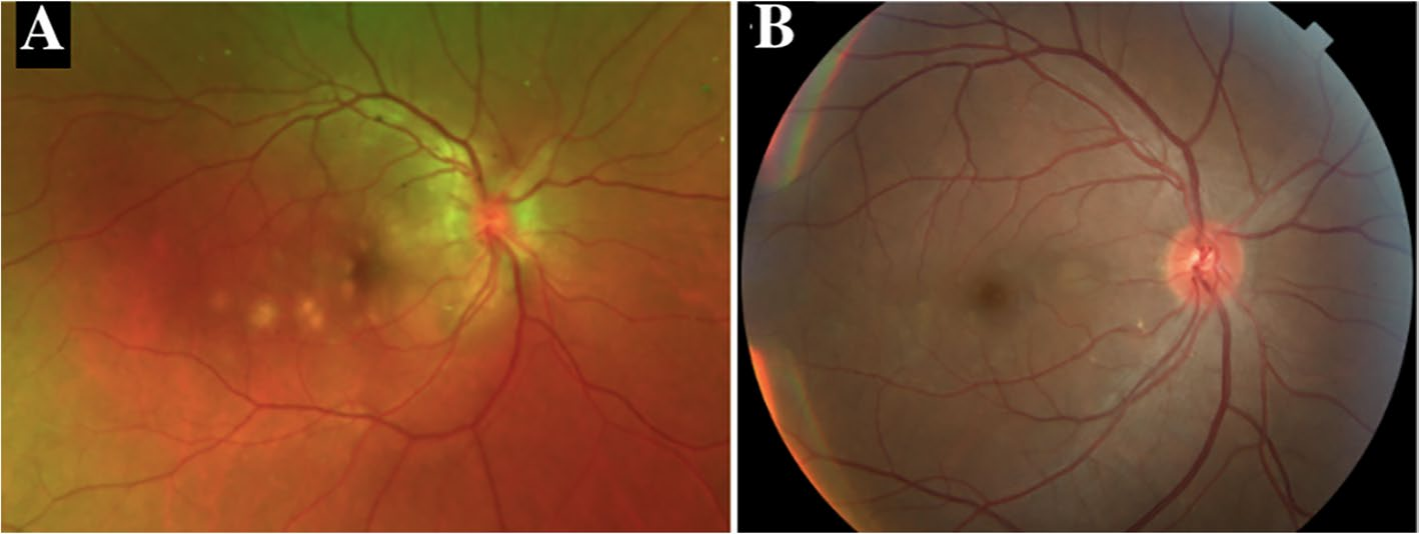
**a** Color fundus photograph (CFP) of the right eye demonstrating parafoveal placoid lesions. **b** CFP of the right eye demonstrating resolution of parafoveal placoid lesions after treatment with oral prednisone

**Fig. 2:**
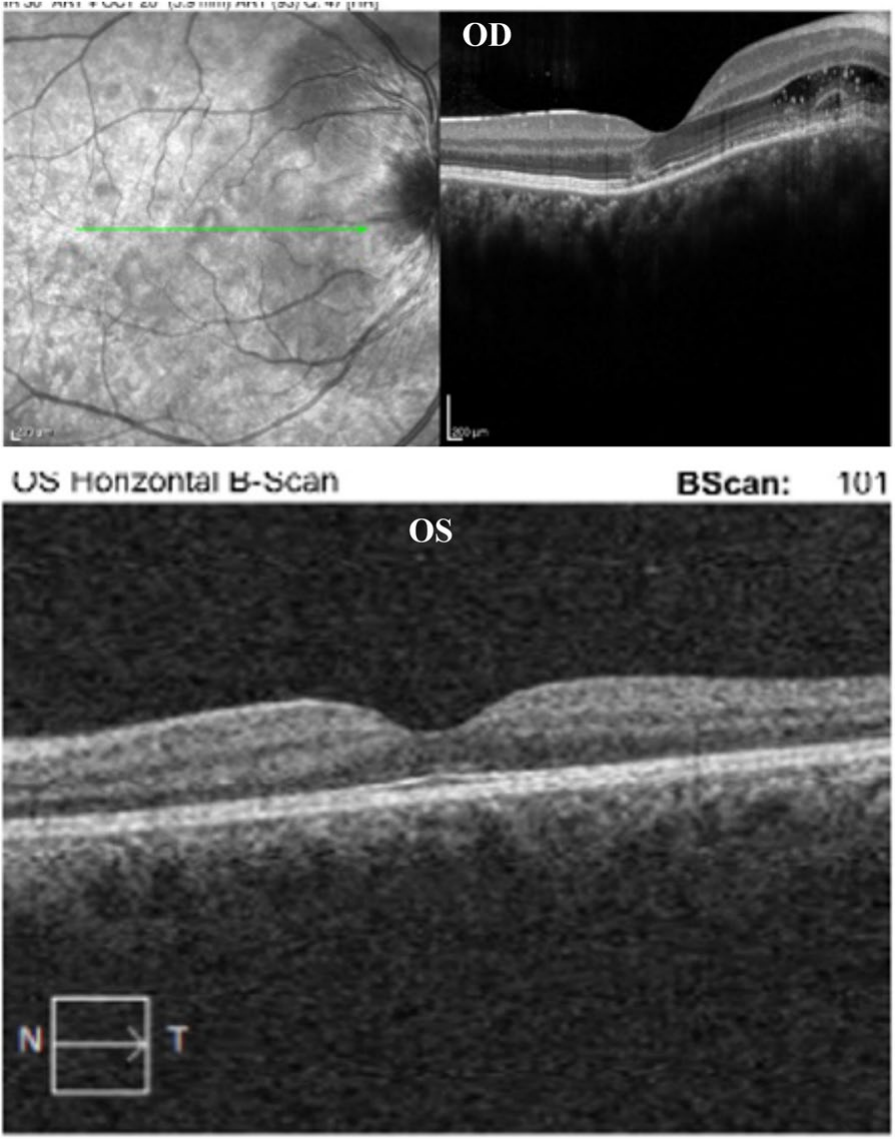
Optical coherence tomography (OCT) of the right eye (OD) showing outer retinal hyper-reflectivity, attenuation of the ellipsoid zone, and intraretinal fluid tracking from the optic nerve. OCT of the left eye (OS) is notable for normal appearance

Best corrected visual acuity (BCVA) was 20/25–2 OD and 20/20–1 in the left eye (OS). Slit lamp examination revealed 0.5 + cells in the anterior chamber (AC) OD. Dilated fundus examination (DFE) revealed a hyperemic disc with blurred disc margins OD. Several creamy, yellow-white, placoid, parafoveal lesions were identified on examination and noted on color fundus photography (CFP) OD (Fig. 1a). Optical coherence tomography (OCT) demonstrated outer retinal hyper-reflectivity and attenuation of the ellipsoid zone as well as intraretinal fluid tracking from the right optic nerve (Fig. 2). The left eye examination and multimodal imaging were within normal limits (Fig. 2).

**Fig. 3:**
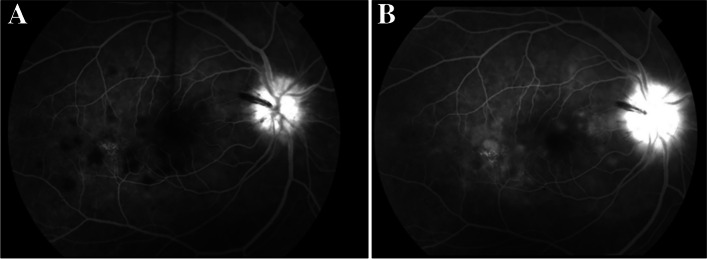
**a** Fluorescein angiography (FA) of the right eye demonstrating early hypofluorescent staining of placoid lesions. **b** FA of the right eye demonstrating late hyperfluorescent staining of placoid lesions

**Fig. 4:**
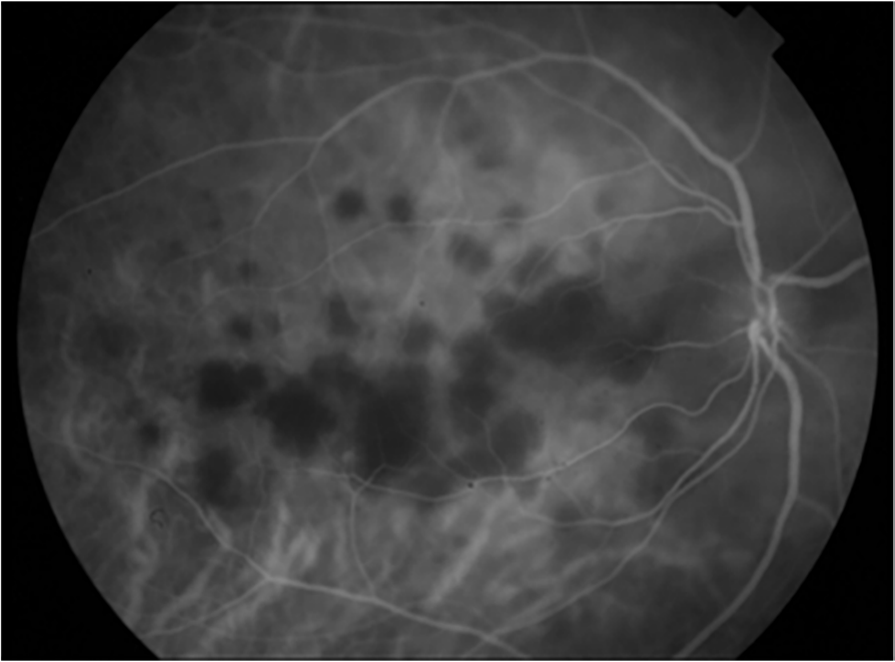
Indocyanine green angiography (ICGA) showing hypofluorescent staining of placoid lesions in the right eye

FA demonstrated early hypofluorescence (Fig. 3a) with late hyperfluorescent (Fig. 3b) staining of the placoid lesions OD. ICGA showed more hypofluorescent areas than were noted on FA alone (Fig. 4). These patterns on FA and ICGA were consistent with the diagnosis of APMPPE. Limited serologic workup revealed a normal complete blood count with differential, negative Treponema pallidum antibody, and negative Quantiferon Gold. The patient underwent an MRI and MRA of the brain which were negative for cerebral vasculitis. The patient was started on 50 mg oral prednisone with a weekly taper with symptomatic improvement and clinical resolution of both optic nerve edema and placoid macular lesions of the right eye (Fig. 1b).

**Fig. 5:**
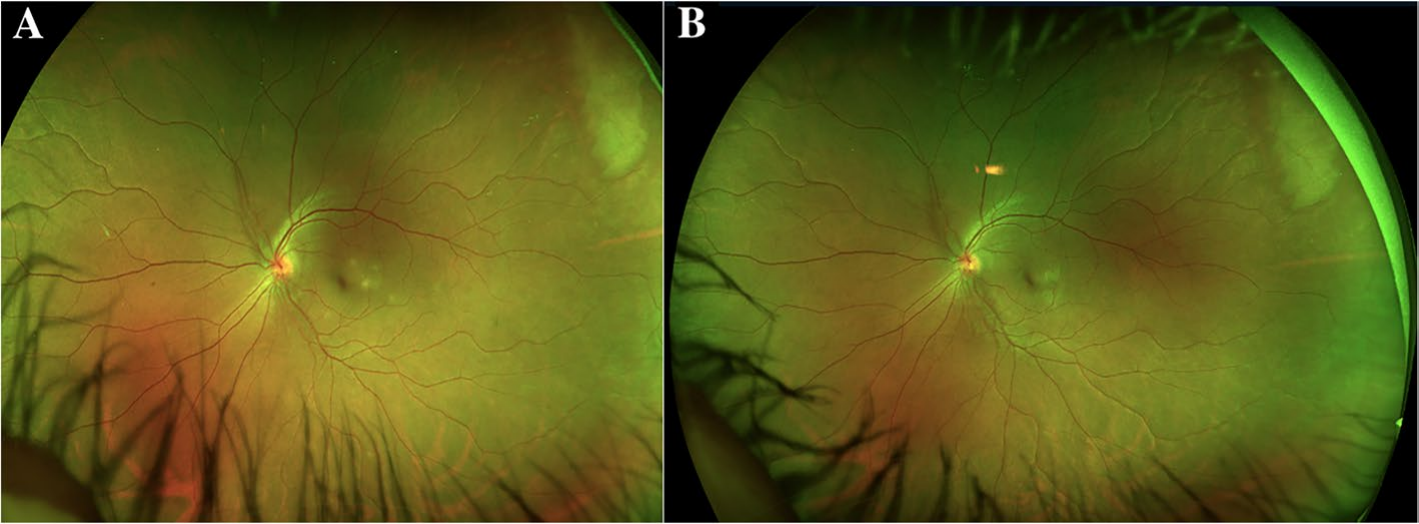
**a** CFP of the left eye showing parafoveal placoid lesions. **b** CFP of the left eye showing resolution of parafoveal placoid lesions after treatment with oral prednisone

**Fig. 6:**
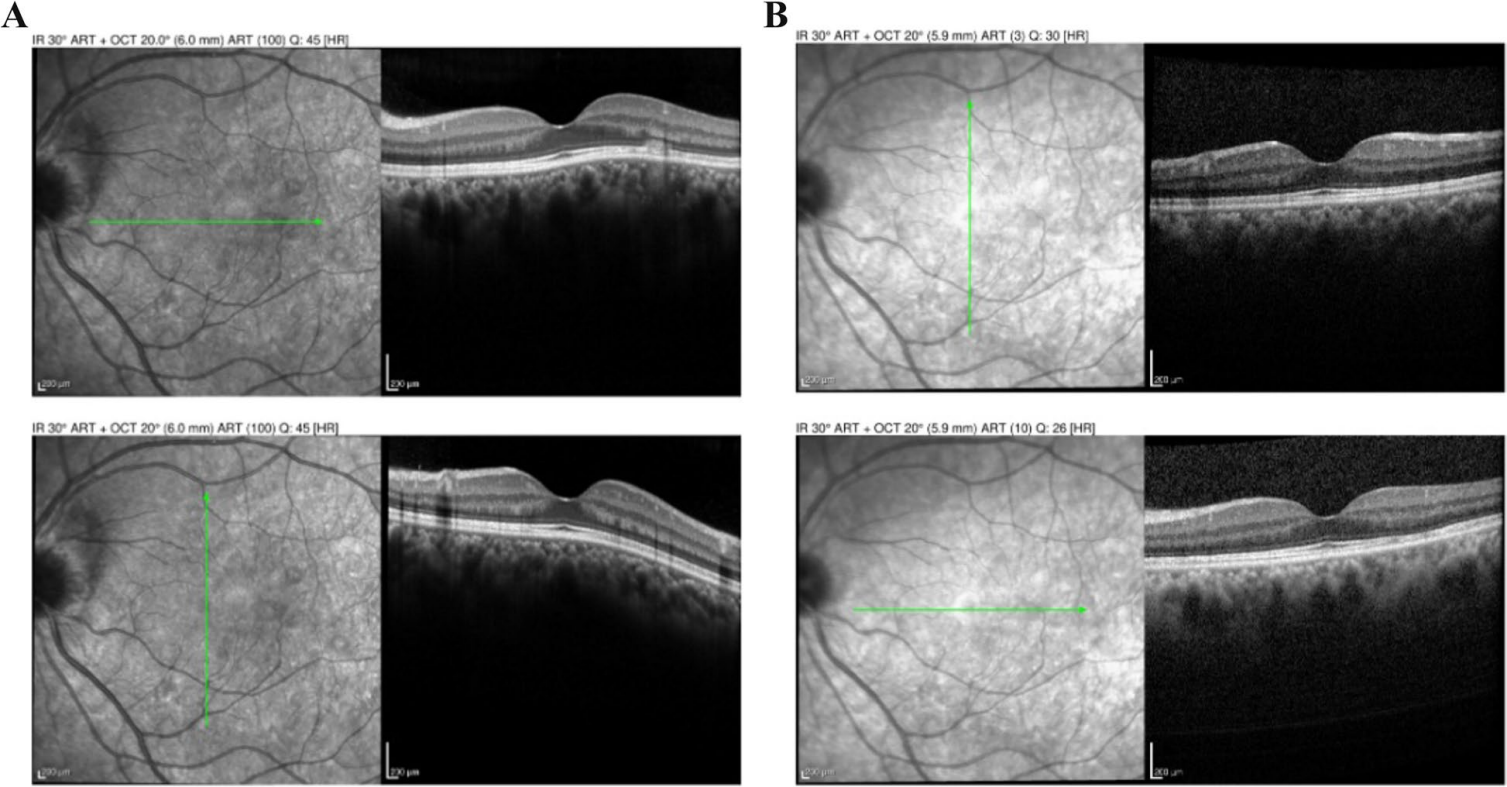
**a** OCT of the left eye showing outer retinal hyper-reflectivity and attenuation of the ellipsoid zone temporal to the fovea. **b** OCT of the left eye showing significant improvement after treatment with oral prednisone

**Fig. 7:**
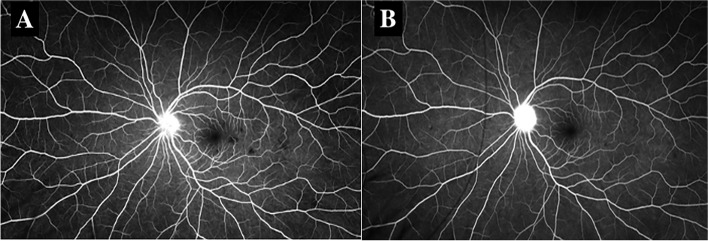
**a** and **b** FA demonstrating early hypofluorescent staining of placoid lesions in the left eye

**Fig. 8:**
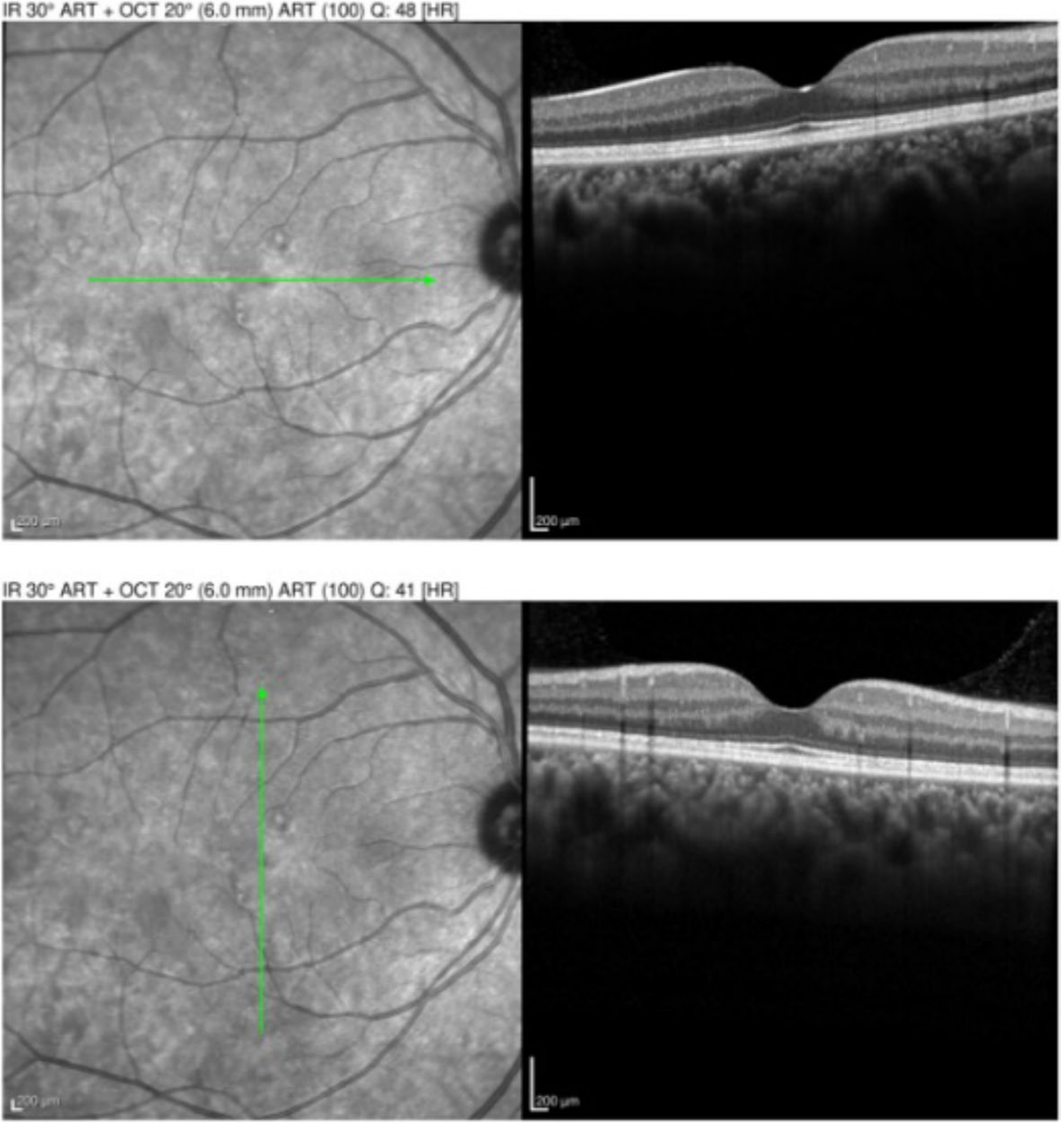
OCT of the right eye is notable for normal appearance

31 months later the patient presented as an emergency visit for similar symptoms of new onset blurry vision in the left eye. The patient denied any photopsias or other ocular or neurologic symptoms. She endorsed receiving an influenza vaccine one month prior to her symptoms. BCVA was 20/20–1 OD and 20/20–1 OS. Examination revealed a quiet AC in both eyes and a normal DFE in the right eye but was significant for mild disc edema and multiple creamy, yellow-white placoid lesions temporal to the fovea of the left eye (Fig. 5a). OCT showed outer retinal hyper-reflectivity and attenuation of the ellipsoid zone temporal to the fovea (Fig. 6a). FA revealed early hypofluorescence of the lesions in the left eye (Fig. 7a, b). OD was within normal limits on exam and multimodal imaging (Fig. 8). Given the patient’s history and clinical findings, the diagnosis of recurrent APMPPE in the contralateral eye was made. Repeat syphilis testing returned negative. She was once again started on oral prednisone with significant improvement in symptoms. Follow-up examination demonstrated improvement in vision and resolution of left optic nerve edema and placoid lesions in the macula (Fig. 5b, Fig. 6b). The patient deferred repeat MRI/MRA testing for evaluation for CNS vasculitis.

## Discussion

This patient’s acute onset blurry vision, photopsias, and peripheral distortion in the setting of macular placoid lesions is most consistent with APMPPE, an inflammatory chorioretinopathy. Patients often present with acute vision loss that may be associated with central or paracentral scotomas, photopsias, and/or metamorphopsia [6]. Atypical APMPPE is likely in this patient who had unilateral involvement and contralateral vision symptoms occurring > 6 months later, with subretinal fluid and papillitis that improved with steroid treatment. Her first and second presentations were preceded by a viral prodrome and influenza vaccination, respectively, both of which have been associated with APMPPE [2, 6]. Coxsackievirus B4 and Adenovirus type 5 have been implicated [7]. FA and ICGA imaging showed staining patterns characteristic of APMPPE. Choriocapillaris hypoperfusion secondary to inflammation has been hypothesized to cause the early phase pattern [2, 6], while late hyperfluorescence may be due to vascular leakage [8]. Hypofluorescence on ICGA also reflects choriocapillaris hypoperfusion, supporting APMPPE as a primary choroidal vasculitis with secondary involvement of the RPE [9].

While the exact etiology of APMPPE is unclear, there are several proposed mechanisms. APMPEE was first described by Gass in 1968, who hypothesized that outer retinal inflammation resulted in placoid lesions at the level of the RPE [1]. With the advent of FA and ICGA imaging, the prevailing theory now describes APMPPE as an inflammatory vasculitis of the choroid, leading to hypoperfusion and ischemic injury of the RPE with subsequent lesion formation [9]. The temporal association of APMPPE with viral illnesses and certain vaccinations suggests that choroidal inflammation may stem from infectious or immune-driven processes. A vasculitic pathophysiology is further supported by the presence of APMPPE in patients with comorbid systemic vasculitis such as cerebral vasculitis and erythema nodosum [9].

Creamy placoid lesions are not unique to APMPPE and have been found in other white dot syndromes such as serpiginous choroidopathy (SC) and relentless placoid choroiditis (RPC). SC typically presents with unilateral central vision changes that progress to involve the other eye, which was seen in this patient. However, placoid lesions originate in the peripapillary area and expand in a serpentine fashion, while our patient’s lesions were centered on the macula and did not exhibit such progression [10]. RPC, also known as ampiginous choroiditis, represents chronic APMPPE that persists for more than 6 months. It is characterized by numerous placoid lesions (> 50) around the fundus and is unlikely in this patient who presented with only a few placoid lesions with resolution.

APMPPE is usually bilateral at onset, with only a few unilateral cases described in the literature and only a smaller handful of them progressing to involve the fellow eye. Li et al. conducted a retrospective case series of seven patients diagnosed with APMPPE, only one of whom had unilateral presentation with contralateral involvement two days later [7]. One month after treatment with IV solumedrol for three days and a slow taper of prednisone, the patient had no evidence of active disease. Another retrospective case series described three patients with unilateral APMPPE. One patient developed contralateral eye involvement four days later, with steroid treatment resulting in lesion resolution ten months later [11]. Kutluturk et al. conducted a retrospective case series of eleven patients with unilateral APMPPE, only two of which developed contralateral involvement. The fellow eye involvement time in these two cases was one and four months after initial presentation [12].

A prospective case series of eight patients with APMPPE revealed three unilateral cases that had subsequent contralateral lesions. The longest time between initial onset and fellow eye involvement was eleven weeks [13]. In a case report by Nakajima et al., unilateral APMPPE self-resolved two months after onset, and fundus exam did not reveal any recurrence in either eye on annual follow up [14]. Most recently, unilateral APMPPE with no contralateral involvement was identified in a COVID-19 patient [15].

## Conclusions

APMPPE is a rare choroidal vasculitis, usually resulting in bilateral central vision changes on initial presentation. Single eye involvement with delayed contralateral presentation, as seen in this patient, is even more uncommon. Unilateral APMPPE has been noted in several patients [7, 11–15]. To the best of our knowledge, the patient described herein has the longest documented time of 31 months between initial onset of placoid lesions and fellow eye involvement. This case demonstrates that unilateral lesion resolution following steroid treatment can still be followed by contralateral eye involvement years later, underscoring the importance of routine ophthalmic monitoring for patients with unilateral APMPPE. Our case further highlights that APMPPE is on a spectrum of disease and although there is a classic presentation, there are many cases such as this one that do not fit the classic description.

## Data Availability

All data generated or analysed during this study are included in this published article.

## Abbreviations

APMPPE: Acute Posterior Multifocal Placoid Pigment Epitheliopathy
RPE: Retinal Pigment Epithelium
OD: Right Eye
OS: Left Eye
FA: Fluorescein Angiography
ICGA: Indocyanine Green Angiography
BCVA: Best Corrected Visual Acuity
AC: Anterior Chamber
DFE: Dilated Fundus Exam
CFP: Color Fundus Photography
OCT: Optical Coherence Tomography
SC: Serpiginous Choroidopathy
RPC: Relentless Placoid Choroiditis
